# Association of pedigree indexes and genomic breeding values with the performance of Polish Holstein–Friesian cows

**DOI:** 10.1007/s13353-024-00921-9

**Published:** 2024-11-15

**Authors:** Tomasz Strabel

**Affiliations:** Department of Genetics and Animal Breeding, Poznan University of Life Sciences, Poznan, Poland

**Keywords:** Breeding value, Dairy cows, Genotype by environment interaction, Genomic selection, Genotyping, Pedigree indexes

## Abstract

Pedigree indices (PI) and genomically enhanced breeding values (GBV) of Polish Holstein–Friesian heifers were compared with their future performance. Phenotypes of 15,794 cows from 294 herds were analyzed. The traits evaluated included milk, fat and protein yield, somatic cell score (SCC), stature, overall udder and feet and legs score, heifer conception rate, and longevity. PI and GBV were from official evaluation systems, and performance records were adjusted for non-genetic effects. Correlations between breeding values and adjusted phenotypes were analyzed. Additionally, cows within each herd were divided into quartiles based on their breeding values, and the performance of cows from the top and bottom quartiles was compared. For production traits, similar analyses were conducted separately for high and low milk-producing herds to check for possible genotype by environment interaction. The analyses confirmed the significant correlation between breeding values and cow performance. Incorporating genomic information into PI significantly improved the predictive accuracy for milk, fat, and protein yields, as well as for SCC and stature, with increases ranging from 34 to 64%. Comparisons of cows’ performance from the top and bottom quartiles supported these observations for all these traits except protein yield, with differences in performance being 43–92% greater when cows were ranked by GBV instead of PI. In a more productive environment, greater differences in performance were found between the top and bottom quartiles. These findings suggest that Polish breeders can enhance the outcomes of their breeding decisions by using GBV instead of PI. This change offers particular benefits in improving the predictability of future performance for the most economically important traits such as milk yield, fat yield, protein yield, somatic cell score, and stature.

## Introduction

The genetic progress in dairy cattle was for a long time generated by the use of semen of proven bulls which were evaluated with high accuracy. Genomic selection, which relies on the selection of younger animals with lower accuracy, was widely introduced in dairy cattle breeding, particularly in the Holstein breed, in 2009 and increased the speed of genetic improvement (Guinan et al. 2023). Initially, the extent to which breeders would adopt this new technology and base their selection decisions on genomic breeding values was uncertain. A similar situation took place during the introduction of the MOET breeding program which also relied on the selection of unproven bulls (Colleau and Mocquot 1989), although the implementation of the MOET programs, in general, was not successful, with genomic selection the rate of adoption proved to be rapid. In the USA, within 3 years of the official implementation of genomic evaluation, 48% of bulls used for artificial insemination were young bulls that had undergone genomic testing. This proportion increased to 73% 10 years after the first genomic breeding values (GBV) were officially published (Wiggans and Carrillo 2022). Following the genomic selection of bulls, genotyping of females followed—reducing the size of microarrays decreased the cost which made their use more affordable. With the advancement of genomic selection, the use of sexed semen has become more widespread. The use of sexed semen, combined with the genotyping of young females, allows for the production of predominantly female offspring of higher genetic merit by making it possible to select the mothers of future replacements (Weigel et al. 2024). By doing so, the dam-to-daughter path of selection is explored more effectively (Bouquet and Juga 2013). The additional benefits of genotyping females include parentage verification and the discovery of unknown parents, the avoidance of mating carriers of the same recessive conditions, and improved management of inbreeding based on actual (molecular) rather than statistical inbreeding coefficients (Hjortø et al. 2015; Ettema et al. 2017). All of this should ultimately result in economic gains for breeders (Calus et al. 2015).

Although genomic selection is no longer a novel tool, its application on the female side varies significantly across different countries. For instance, in Australia, only 4% of herd-recorded cows are genotyped (DataGene 2020), whereas the Council on Dairy Cattle Breeding reports a 22% genotyping rate in the USA (USCDCB 2020). The varying adoption rates of new technologies may be attributed to country-specific factors and are the subject of ongoing research (von Keyserlingk et al. 2024). For a breeder to decide to genotype females, the motivation must surpass any potential skepticism. The breeder must be convinced that the new technology is effective and that the financial investment will be worthwhile. Additionally, they must be prepared to commit the necessary effort to collect biological samples and address any potential issues related to pedigree inconsistencies. The cost of genotyping and the waiting time for results are also important (Newton and Berry 2020). While multiple factors may contribute to the slower adoption of new technologies, it is essential that the use of female genotyping is justified by tangible production benefits. These benefits depend on how well the GBV of heifers can predict their future performance, which is critical for making informed breeding decisions.

The accuracy of breeding value estimation is crucial for the selection and use of sires. For traditional breeding value estimation of bulls, accuracy primarily depends on the number of daughters, the number of herds in which these daughters perform, and the heritability of the trait. The accuracy of GEBV, however, is influenced by additional parameters, notably the size of the reference population and the heritability of the trait (Goddard and Hayes 2009). Initially, predicting the practical performance of bulls selected primarily based on their genotype was challenging. Over time, the performance of their daughters has validated that GBVs are suitable for selection, which was consistent with the results obtained using officially approved methods (Mäntysaari et al. 2010).

The use of genomic predictions is preceded by a thorough validation process (Mäntysaari et al. 2010). In dairy cattle, validation typically focuses on bulls, comparing their GBV obtained at an early age with breeding values estimated based on the performance of numerous daughters, thereby achieving a high accuracy. The statistical methods currently employed for this kind of evaluation are described by Legarra and Reverter (2018) and primarily stem from linear regression analysis. Such validation should be continuous, as it is essential for enhancing the quality of evaluations. This ongoing process can identify traits that warrant further investigation and provide valuable insights into the practical application of genomic information in breeding programs.

The validation process for female GBV often uses a slightly different approach—GBV obtained for heifers are typically compared with their own future recorded performance (Weigel et al. 2015; Bengtsson et al. 2020; Toghiani et al. 2024). Validation based on female records provides breeders with a more applicable analysis, as it can be performed directly on trait values used in herds to describe cow performance, expressed in trait-specific units rather than in standardized breeding value points. In the case of validating females’ proofs, using performance records collected from farms where genotyping was utilized can increase trust in the results of breeding value estimation.

Cattle breeding in Poland primarily focuses on the Polish Holstein–Friesian breed, which comprised 693,558 cows under milk recording in 2023 (PFHBiPM 2024). The genomic selection was officially introduced in Poland in 2014, following the country’s entry into a European consortium in 2009 that facilitated access to a large reference population by providing genotypes from thousands of bulls. Despite the availability of this advanced technology, Polish farmers have exhibited a relatively low interest in adopting advanced and precise breeding techniques, with only 38% of cows participating in the official milk recording program (PFHBiPM 2024). While there has been a gradual increase in the interest in female genotyping, its adoption remains limited. In 2022, more than 20,226 females were genotyped in the main laboratory, accounting for less than 3% of cows enrolled in the milk recording program (PFHBiPM 2023).

Despite these limitations, dairy cattle breeding in Poland has seen significant progress in recent years. Average milk production per cow under milk recording increased from 6664 kg in 2006 to 9150 kg in 2023, while the number of cows remained stable (PFHBiPM 2024), indicating improvements in both management practices and genetics. As average milk production has risen, this may have led to increased variation in milk yield among herds, potentially giving rise to possible genotype-by-environment (GxE) interaction.

The existence of GxE interactions complicates animal breeding by making the prediction of an animal’s performance more difficult. When GxE interactions are present, differences between genotypes observed in various environments are not consistent, and in extreme cases, the ranking of animals may change. A common approach to analyze GxE is to use multiple-trait models in genetic evaluations, treating traits measured in different environments as distinct but correlated traits. As reviewed by Hayes et al. (2016), a genetic correlation between two traits below 0.8 is generally considered low, suggesting that the traits measured in different environments are not genetically equal. This method is routinely used by Interbull (2024), which compares the breeding values of dairy bulls across different populations, usually from different countries with varying management strategies and even from different continents.

Genetic correlations between the same traits for different countries, as estimated by Interbull, can drop significantly below 0.8 (Powell et al. 2005). However, these correlations are based on breeding values obtained from various populations, each with its own breeding program. Such variability can arise from differences in environmental conditions, trait definitions, data size and structure, and the models and methods used for genetic evaluation. The presence of GxE interaction may also lead to a situation where the performance advantage of animals with higher genetic merit is less pronounced in suboptimal environments (Zwald et al. 2003). This can reduce the likelihood that breeders in lower-productivity herds will adopt intensive selection practices and utilize more advanced genetic tools.

The objective of this study was to assess the predictive ability of pedigree indexes and genomic breeding values for heifers by comparing these values with the animals’ future performance. Additionally, the study aimed to determine whether this predictive accuracy differs between high and low milk-producing herds.

## Material and methods

Data available for this study consisted of genotypes, breeding values, and performance records of 19,564 cows born in the years 2014–2018, and their performance records were collected as a part of the routine milk recording system. Observations came from 1201 herds, and 12,818 cows had been scored for conformation. All the available cows had completed their first lactations.

All the animals were required to be genotyped before their first performance records were available for their genetic evaluation, which means no later than 14 months of their age. As a consequence, their first genomic breeding values were available between 2016 and 2019. At this period of time, it was not common to genotype all the heifers in the herd; hence, only herds with a minimum of four genotyped females were included in the analysis. After edits, records from 15,794 cows present at 294 herds were considered. The average first lactation milk yield in these herds was 9629 kg, with a range of 5216 to 12,945 kg. For fat yield, the values ranged from 215.6 to 526.6 kg, with an average of 372.9 kg. Protein yield ranged from 175.7 to 449.7 kg, with an average of 325.1 kg. For heifer conception rate (HCR), within-herd variation greater than 0 of the number of inseminations was required, which consequently reduced the number of records available for this trait.

Genomic breeding values (GBV) and pedigree indexes (PI) for the females were obtained from the Center of Genetics at the Polish Federation of Cattle Breeders and Dairy Farmers. The GBV and their reliabilities were official breeding values calculated by the National Research Institute of Animal Production. In the case of GBV, a two-step approach is employed (NRI 2024). Direct genomic values (DGV) were estimated based on SNP effect solutions using a mixed model that includes a random polygenic effect. GBV is calculated as a combination of an individual’s pedigree index and DGV, using selection index theory. Pedigree indices and their reliabilities were calculated by the Center of Genetics (CGen 2024a), with pedigree indices derived from the breeding values of four male ancestors in each female’s pedigree, and reliabilities calculated from the reliabilities of these four male ancestors using selection index theory. At the time of data collection for this study, these indexes were not replaced with parent averages, even if both parents had been genotyped.

A total of nine traits were analyzed in this study: three production traits (milk yield, fat yield, and protein yield), three conformation traits (stature, overall udder score, and feet and legs), and three functional traits (somatic cell count, heifer conception rate, and longevity). The heritabilities for these traits are presented in Table 1.

**Table 1:** Heritability, reliability of pedigree indexes (PI) and genomic breeding values (GBV), and corresponding phenotypes for the analyzed traits

Trait	Heritability	Reliability of PI^a^	Reliability of GBV^b^	Relative increase in reliability [%]^c^	Observed performance
Milk yield [kg]	0.33	0.32	0.71	122	First 305d lactation milk yield [kg]
Fat yield [kg]	0.29	0.32	0.71	122	First 305d lactation fat yield [kg]
Protein yield [kg]	0.29	0.31	0.71	129	First 305d lactation protein yield [kg]
Somatic Cell Score [points]	0.32	0.32	0.67	109	Average first 305d lactation somatic cell score [thousands in ml]
Stature [points]	0.54	0.32	0.75	134	Stature (cm)
Feet and legs [points]	0.11	0.28	0.37	32	Feet and legs score [points]
Udder [points]	0.14	0.29	0.54	86	Udder score [points]
Heifer conception rate [points]	0.03	0.26	0.36	38	Number of inseminations to first conception
Longevity [points]	0.19	0.32	0.54	69	Survival until the second lactation

^a^Standard deviation of PI reliabilities equaled 0.02

^b^Standard deviation of GBV reliabilities equaled 0.03

^c^Relative increase in reliability was calculated as the difference in reliabilities of PI and GBV in relation to PI reliability

The genomic breeding values (GBV) used were the first values obtained following genotyping. Although these breeding values were derived from several routine evaluations, they were analyzed together because the genetic base was consistently defined for all traits. In Poland, the genetic base for calculating relative breeding values is updated every 5 years.

It is important to note that while breeding values correspond to phenotypes, they are not always defined in the same way (see Table 1). Breeding values for production traits and SCC are based on data from the first three lactations, whereas the corresponding analyzed phenotypes were from the first lactation only. For SCC breeding values, the scale is reversed so the higher the breeding value, the higher resistance to mastitis should be observed and the lower number of somatic cell count in milk. Breeding values for heifer conception rate, calculated based on the number of insemination till the successful one, were compared with the number of inseminations required for heifers. For these traits, a higher breeding value means a lower number of inseminations should be required to get a cow pregnant. Longevity breeding values, defined as a length of productive life measured by the number of days from first calving to culling or censoring, were compared with survival until the second calving. Only for the conformation traits were the females’ breeding values directly compared with their phenotypic scores.

To verify the predictive ability of genomic breeding values, available phenotypes were adjusted using a mixed model in the SAS software version 9.4 (SAS Institute Inc., Cary, NC). For this purpose, the following linear model was employed:

$${y}_{\text{ijkl}}={\text{AGE}}_{\text{i}}+{\text{HERD}}_{\text{j}}+{\text{YC}}_{\text{k}}+{\text{MC}}_{\text{l}}+{e}_{\text{ijkl}}$$

where *y*_ijkl_ is the first lactation performance of the cow, AGE_i_ is the fixed effect of the *i*-th age of the calving class, HERD_j_ is the fixed class effect of herd *j* in which a cow was located, YC_k_ is the fixed class effect of calving year *k*, MC_l_ is the fixed effect of l month of calving, and *e*_ijkl_ is a random residual which was assumed to have normal distribution *N*(0,${\sigma }_{e}^{2}$). The following age classes were defined based on months at calving: under 23 months; separate classes for 24, 25, and 26 months; and over 26 months. The effects of the year of calving and month of calving were defined differently from the national evaluation model due to the smaller number of records used in this analysis. Residuals from this model were used as adjusted phenotypes for subsequent comparisons with individual GBV.

The correlation between breeding values (PI and GBV) and between PI or GBV and adjusted phenotypes was calculated using Pearson’s correlation. To assess the significance of differences between these correlations, a 95% confidence interval was constructed using Fisher’s *Z* transformation. To compare the performance of cows with the highest and the lowest genetic merit, females were categorized into four quartiles based on PI or GBV across different herds. To compare the average differences between the adjusted phenotypes of females in the lowest and highest quartiles of PI or GBV across herds, Cohen’s *d* effect size was computed (Cohen 1988). The 95% confidence intervals for Cohen’s *d* were also determined.

To construct the confidence intervals for Cohen’s *d* values, the following formula was employed:

$$CI=d\pm (Z\times {SE}_{d})$$

where *Z* is the value from the standard normal distribution for the selected confidence level and SE_d_ is a standard error for Cohen’s *d* calculated using the following formula:

$${SE}_{d}=\sqrt{\frac{{n}_{1}+{n}_{2}}{{n}_{1}{n}_{2}}+\frac{{d}^{2}}{2({n}_{1}+{n}_{2})}}$$

where *n*_1_ and *n*_2_ equaled numbers of records in the compared quartiles.

The presence of genotype by environment interactions was assessed by applying the aforementioned methods to high and low production herds separately. The herds were categorized into two groups based on their average milk yield, which was 9601 kg. As the average number of genotyped females per herd in lower-producing herds was three times lower (approximately 5 vs. 15), this led to a smaller number of analyzed females from this group of herds.

## Results

The correlations between PI and GBV were moderate, ranging from 62.9 to 63.0% for production traits, 54.5 to 62.9% for type traits, and 32.5 to 58.4% for functional traits (Table 2). The absolute values of correlations between breeding values and adjusted phenotypes ranged from 2 to 28% for PI and from 4 to 42% for genomic breeding values and were all statistically significant. It is important to note that, due to the different definitions of breeding values and performance traits for SCC, longevity, and HCR, negative correlations are desired. For PI, the highest correlations were observed for production traits and stature (above 20%), while the lowest was for longevity, HCR, and feet and legs (below 10%). Overall, the correlations between breeding values and adjusted phenotypes were higher than those between PI and adjusted phenotypes, with statistically significant differences observed only for production traits, stature, and SCC. The most significant relative change in correlation was noted for fat yield and SCC (64%), while the smallest was for protein yield (34%).

**Table 2:** Correlations between adjusted phenotypes and both pedigree indexes (PI) and genomically enhanced breeding values (GBV); *N* denotes the number of females analyzed for each trait

Trait	*N*	Correlation with PI	Correlation with GBV	Relative change in correlation [%]^a,b^
Milk yield	11,847	0.23	0.33	**46**
Fat yield	11,847	0.19	0.31	**64**
Protein yield	11,847	0.19	0.25	**34**
Stature	11,233	0.28	0.42	**50**
Udder	11,233	0.13	0.14	9
Feet and legs	11,233	0.09	0.11	27
SCC	11,838	− 0.14	− 0.23	**64**
Longevity	11,846	− 0.02	− 0.04	36
Heifer conception rate	6403	− 0.05	− 0.04	− 23

^a^The difference between correlation statistically significant (*P* < 0.05) is in bold

^b^Relative change was calculated as the difference in correlations of adjusted phenotypes with PI and GBV in relation to the correlation between adjusted phenotype and PI

The average differences in adjusted cow phenotypes between the top and bottom quartiles of herds were significantly greater when quartiles were assigned using GBV instead of PI for the following traits: milk yield, fat yield, somatic cell score, and stature (Table 3). For these traits, the relative change in differences was the highest for SCC (78%), followed by fat yield (64%) and milk yield (62%). The ratio of the average difference in adjusted performance for GBV to the average difference for PI was 0.58 for milk yield and 0.51 for fat yield, indicating that one unit of PI for these traits corresponded to slightly more than 0.5 units of performance. Incorporating genomic information into breeding value estimation increased these ratios to 0.81 and 0.82 for milk yield and fat yield, respectively. For stature and SCC, these ratios were much smaller, which is a consequence of the different scales in which breeding values and observed units are expressed. The values of Cohen’s *d*, measuring the effect size (which in this context quantifies the difference between the top and bottom quartiles), were generally lower for traits where there was no significant difference between the effect sizes when using GBV instead of PI (ranging from 0.1 to 1.1). In cases where the differences in effects were significant, Cohen’s *d* values for GBV were very high, ranging from 1.1 to 1.9.

**Table 3:** Average differences in adjusted cow phenotypes between herd top and bottom quartiles defined by pedigree indexes (PI) or genomic enhanced breeding values (GBV) and the ratio between the average difference in PI or GBV and average differences in adjusted cow phenotype for females top and bottom quartiles associated with Cohen’s *d*

Trait	Average differences of the adjusted cow phenotypes between herd top and bottom quartiles		Relative change in in average differences^a^ [%]	Ratio between the average difference in PI or GBV and average differences in adjusted phenotype		Cohen’s d for average differences^b^	
	PI	GBV		PI	GBV	PI	GBV
Milk yield	363.9	600.9	64	0.58	0.81	− **1.1**	− **1.5**
Fat yield	12.81	22.60	76	0.51	0.82	− **0.8**	− **1.4**
Protein yield	9.40	12.84	37	0.52	0.69	− 0.8	− 1.0
Stature	1.16	1.65	43	0.09	0.13	− **1.4**	− **1.9**
Udder	0.83	0.82	− 1	0.07	0.07	− 0.7	− 0.9
Feet and legs	0.31	0.57	86	0.02	0.04	− 0.3	− 0.6
SCC	− 0.23	− 0.45	92	− 0.02	− 0.03	**0.5**	**1.1**
Heifer conception rate	− 0.05	− 0.04	− 25	0.00	0.00	0.2	0.2
Longevity	− 0.01	− 0.01	7	0.00	0.00	− 0.1	− 0.1

^a^Relative change calculated as the difference in average differences of the adjusted cow phenotypes between herd top and bottom quartiles defined with PI and GBV in ratio to the average difference for PI

^b^Significant differences in Cohen’s *d* are in bold

When correlations between PI or GBV and adjusted phenotypes were calculated for production traits separately for high and low milk-producing herds, the estimates were lower for low-producing herds than for high-producing herds (Table 4). Adding genomic information led to a statistically significant increase in relative changes in correlations for all analyzed traits. The pattern of increase was not consistent across traits: the relative increase in correlation was higher for milk yield in high-producing herds compared to low-producing herds, whereas for protein and fat yield, a greater increase in correlations was observed in low milk-producing herds.

**Table 4:** Correlations between adjusted phenotypes and heifer pedigree indexes (PI) as well as genomically enhanced breeding values (GBV) for low and high milk-producing herds; *N* denotes the number of females analyzed for each trait

Trait	*N*	Correlation with PI	Correlation with GBV	Relative change in correlation^a,b^
Milk yield				
Low	2920	0.19	0.30	**0.61**
High	8927	0.25	0.35	**0.40**
Fat yield				
Low	2920	0.17	0.31	**0.65**
High	8927	0.19	0.31	**0.63**
Protein yield				
Low	2920	0.15	0.23	**0.56**
High	8927	0.20	0.27	**0.28**

^a^The difference between correlation statistically significant (*P* < 0.05) is in bold

^b^Relative change was calculated as the difference in correlations of adjusted phenotypes with PI and GBV in relation to the correlation between adjusted phenotype and PI

When differences between adjusted phenotypes from the top and bottom quartiles were calculated for production traits separately for high and low milk-producing herds, they were found to be higher for high-producing herds compared to low-producing herds (Table 5). However, the pattern of increase varied across traits, and not all changes were statistically significant. Only for milk yield were the average differences for adjusted phenotypes significantly higher for both low- and high-producing herds. Additionally, significant differences were observed for fat and protein yields in high milk-producing herds. The ratio of the average difference in adjusted performance to the average difference in GBV for all production traits in high-producing herds exceeded 0.8, reaching 0.91 for fat yield.

**Table 5:** Average differences in adjusted cow phenotypes between herd top and bottom quartiles defined by pedigree indexes (PI) or genomic enhanced breeding values (GBV) and the ratio between the average difference in PI or GBV and average differences in adjusted cow phenotype for females top and bottom quartiles associated with Cohen’s *d*. Significant differences in Cohen’s *d* are in bold. Calculations were done separately for low and high milk-producing herds

Trait and production level	Average differences of the adjusted cow phenotypes between herd top and bottom quartiles		Relative change in in average differences^a^ [%]	Ratio between the average difference in PI or GBV and average differences in adjusted phenotype		Cohen’s *d* for average differences^b^	
	PI	GBV		PI	GBV	PI	GBV
Milk yield							
Low	308.6	556.9	80	0.49	0.78	− **0.7**	− **1.3**
High	421.0	643.6	53	0.67	0.85	− **1.1**	− **1.8**
Fat yield							
Low	12.10	18.40	52	0.50	0.68	− 0.7	− 1.1
High	13.54	26.00	92	0.52	0.91	− **0.9**	− **1.7**
Protein yield							
Low	6.70	9.85	47	0.45	0.58	− 0.6	− 0.8
High	12.40	15.70	27	0.61	0.82	− **1.0**	− **1.3**

^a^Relative change calculated as the difference in average differences of the adjusted cow phenotypes between herd top and bottom quartiles defined with PI and GBV in ratio to the average difference for PI

^b^Significant differences in Cohen’s *d* are in bold

## Discussion

Dairy farms have become increasingly reliant on technology, although farmers’ attitudes toward new technology vary (von Keyserlingk 2024). The industry is actively working to increase the adoption rate of new technologies among breeders. Herrick et al. (2024), based on their experience in organizing workshops for producers and veterinarians, found that genomic selection could expand rapidly if producers and their support teams gain access to educational resources that enhance their understanding of the technology’s full potential. Abdelsayed et al. (2022) outlined steps to boost the use of heifer genomic testing in Australia; however, enhancing the correlation between proof and future daughters’ performance was not among them. On the other hand, while genotyping females has the potential to enhance genetic programs, it may not always be cost-effective (Weigel et al. 2012). Newton and Berry (2020) identified conditions under which the return on investment in genotyping heifers is maximized: when there is a substantial difference between the reliability of PI and GBV, when the proportion of selected females to the number of candidates avoids extremes (0–100%), when parentage errors rate is high, when genetic superiority is realized soon after testing (i.e., when the first progeny are born), and when the ratio of the standard deviation of the breeding objective to the cost of genotyping is highest. This underscores that determining the financial benefits of genotyping is a complex task, which may not inherently encourage its adoption. It is assumed that in Poland, the ongoing process of herd enlargement is one of the key contributors to a reduced interest in females’ genotyping.

Obtaining GBVs for females generally requires effort from farmers, along with the costs associated with genotyping and breeding value calculation. An alternative can be parent averages or pedigree indexes, which have been available in Poland for all females. Although their primary purpose is to identify candidates for future bull dams, breeders can also use them for breeding work, despite being less accurate than GBV. Their accuracy is primarily a function of the reliability of breeding values of male ancestors. When breeders make selection decisions based on females’ breeding values, the relatively low correlations between PI and GBV observed in this study (ranging from 32.5 to 63.0) indicate that different animals will likely be chosen, ultimately leading to varying performance outcomes.

Predicting individual cow performance is a complex task that has been approached in various ways, but the effectiveness of these methods is often limited. Generally, the alignment of genomic breeding values with future performance records depends on the accuracy of both sets of information. For breeding values, reliability is crucial and largely depends on trait heritability. Performance records, in addition to reflecting an individual’s genetic merit, rely on the completeness and precision of the collected data. Specifically, gathering data to calculate fertility breeding values can be challenging, and dedicated projects are undertaken to overcome this weakness (Abdelsayed et al. 2017).

The variability of non-genetic factors affecting an individual’s performance is also crucial. Legarra and Reverter (2018) note that when the predictive ability of breeding values is verified using performance records, the sensitivity of the analysis is affected by inaccuracies in heritability estimates and pre-correction for fixed effects. Correcting for non-genetic effects can be particularly challenging when there are numerous levels of primary non-genetic factors and few records within each class, which may have impacted the results of this study. These non-genetic influences were only partially accounted for by the model used in this study. The effectiveness of adjusting performance records for environmental factors was limited by the data structure and size, which do not allow for the removal of non-genetic effects as effectively as during the estimation of breeding values.

The data used in this study was obtained from females that were genotyped and for which performance records were available. Some potential candidates for genotyping may have been culled before tissue collection, and some cows may not have survived until the end of their first lactation. In this type of validation study, it is assumed that these factors do not significantly affect the results. Additionally, animals may have been selectively chosen for genotyping based on various criteria, including poor health status and other indicators of low suitability for breeding, such as individual performance, dam’s performance, sire’s breeding value, or even PI. If the information used for such selection had been accurate, it could have biased the results. Although the specific selection criteria remain unknown (as the data needed to reveal them are not routinely collected), it can be assumed that the information used for this type of selection was not highly accurate. Genotyping of females was conducted at an early age, prior to the availability of individual performance records for the analyzed traits. At the time of genotyping of females included in the analysis, access to pedigree indexes was very limited. Overall, the application of breeding values on Polish dairy farms remains relatively limited. This constraint is evident in the data used for this study, which shows that 42% of bulls are shared between high- and low-producing herds, with 85% of females being sired by these common bulls. Nonetheless, the issue of preselection of females for genotyping may become more prominent in the future as breeders optimize the use of this new tool. Newton and Berry (2020) noted that when only a small number of females need to be selected, genotyping too many animals, including those with a low likelihood of achieving high GBV, may result in diminished profits. On the other hand, a larger number of genotyped females available in the future may increase the precision of this type of analysis.

Overall, the correlations between breeding values and adjusted performance records were lower than the expected square root of heritabilities. This discrepancy arises due to the fact that estimated breeding values, rather than true breeding values, were used, and their reliabilities were clearly less than 100%. The correlations between PI or GBV and first lactation records, ranging from 0.2 to 0.4 in this paper, were somewhat lower than those observed by Bengtsson et al. (2020) for corresponding traits of Holstein, which ranged from 0.25 to 0.40. For milk yield, they found a correlation of 0.31 for the parent average and 0.45 for the GBV with the first lactation record, whereas in this study, these correlations were 0.23 and 0.33 for the pedigree index and GBV, respectively. These differences may arise from the fact that reliabilities for genomic breeding values in this study, ranging from 0.36 to 0.71, were also lower than those obtained by Bengtsson et al. (2020), which did not drop below 0.59 and reached up to 0.77. Although the reliability of genomic breeding values for functional traits was higher in both analyses of Nordic data, the correlation between GBV and longevity performance was the lowest in both analyses, reaching similarly low levels of 2–3%. Toghiani et al. (2024) recently compared genomic breeding values (GBV) with yield deviations in American Holsteins, reporting higher correlations for production traits (0.42–0.50) and similar correlations for somatic cell score (0.25). The reliabilities of GBV for these traits were slightly higher (0.75–0.79) than those observed in this study.

Correlations between breeding values and performance records are influenced not only by the reliability of breeding values but also by the precision and quality of performance records used for comparison, as well as potential discrepancies in trait definitions. For repeated traits, data from consecutive lactations should ideally be used, although this approach requires extended data collection over a longer period. Additionally, breeding values for production traits and SCC reflect performance across the first three lactations, whereas in this study, only the first lactation records were used for comparison. Longevity breeding values encompass the entire lifespan, but here, survival was assessed only up to the second calving. Consequently, the correlation between females’ proofs and their actual performance may be lower than that observed for the correlation between sires’ GBV and the performance of their numerous daughters (Lund et al. 2011).

In the analysis of conformation traits, there were no discrepancies between the definition of breeding value and the analyzed phenotype. The highest correlation (0.42) between GBV and adjusted phenotype was obtained for stature, a trait with high heritability (0.54). However, for other conformation traits with lower heritability, the correlations did not exceed 14%. The reasons for the low correlation between breeding values and performance for heifer conception rate can be attributed not only to the very low heritability of this trait but also to challenges in collecting phenotypic data of good quality. The lack of variability in observed phenotypes across many analyzed herds resulted in 46% fewer observations being included in the analysis of this trait compared to production traits. The reduced data quantity itself may also have contributed to the low correlation observed.

Correlations between breeding values and adjusted performance records significantly increased when GBV replaced PI only for production traits, SCC, and stature. These traits have the highest heritability, ranging from 0.29 (for fat and protein yield) to 0.54 (for stature), while heritabilities for other traits range from as low as 0.03 (HCR) to 0.19 (longevity). Additionally, production traits and SCC are measured with high precision as they are routinely recorded every 4–8 weeks with compliance to ICAR standards, with many single records contributing to the first lactation record. When Bengtsson et al. (2020) compared parent averages of Holsteins from Nordic countries and their GBVs with future adjusted records, the gain in correlation for production traits and SCS was generally comparable to the results of this study. However, the correlations between their breeding values and adjusted records were higher, as were the reliabilities of GBV. Unlike in this study, adding genomic information significantly improved the prediction of future records for fertility, longevity, and other functional traits, as well as for udder and feet and legs. The primary direct cause of this difference lies in the higher reliabilities for non-production traits in the Nordic study, which were not lower than 0.58, whereas in this study, they did not exceed 0.54. For instance, reliabilities for feet and legs and heifer conception rate were 0.37 and 0.36, respectively. In general, traits with low heritability in the study by Bengtsson et al. (2020), such as the interval between first and last insemination, clinical mastitis, calving ease, claw health, and general health, gained relatively more accuracy from using genomic information than highly heritable traits such as production traits. It contrasts with the results presented in this study.

The accuracy of genomic breeding values for functional traits, which typically have low heritabilities, remained low. For production traits, SCC, and stature, the relative increase in reliability of breeding values was the highest, being in the range of 109–129%, substantially higher than for remaining traits (ranging 32–86%). Other authors have indicated that traits in this group particularly benefit from the application of genomic selection (García-Ruiz et al. 2016; Wiggans et al. 2017). The reliability of heifers’ conception rate increased from the reliability of PI by only 32%, reaching 0.36, suggesting that genomic selection has not improved the accuracy of selection for this trait as much as in other countries. Bengtsson et al. (2020) reported an average model reliability of 74% for fertility in Nordic countries. Overall, udder score and feet and legs did not gain on adding genotypes to calculate breeding values when considering predicting future records—similarly to HCR reliability of PI for these traits was below 30% and it did not increase as much as production traits. There were 14 functional traits of Holsteins included in the study of Toghiani et al. (2024), only one of them had a heritability higher than 10%, and for all of them reliability of pedigree-based breeding values at least doubled when genomic information was added.

Longevity, with a moderate heritability of 20%, unexpectedly did not benefit from the inclusion of genomic information in breeding values. Although culling decisions are often made after the first calving and are partially influenced by management strategies (Kargo et al. 2014), the persistent discrepancy between breeding values and observed performance may account for this result. In the study by Bengtsson et al. (2020), a statistically non-significant difference in the correlation between parent average and adjusted performance, and between GBV and adjusted records, was observed for the Jersey breed. In contrast, significant differences were found for Red Dairy Cattle and Holsteins. However, it is important to note that the analysis of longevity in Jerseys included 7053 records, while the other breeds had more than double that number, which could have contributed to the differences in findings. Similarly, Toghiani et al. (2024) reported smaller benefits of using GBV over PI in predicting performance for smaller breeds, such as Ayrshire, Brown Swiss, and Guernsey, compared to the larger Holstein population.

In 2019, an economic index was implemented for the selection of Polish Holstein–Friesian cattle, focusing on traits with the greatest economic importance. This study included all traits considered in the index, except for days open and cow conception rate. These findings confirm that genotyping females can significantly increase the accuracy of breeding decisions; animals retained in the herd based on genotyping results will exhibit better performance than those selected using PI. These differences should translate into improved economic efficiency, as the combined relative importance of milk, fat, and protein yields, as well as SCC and stature, is 74% (CGen 2024b).

Although comparing the performance of cows with low genomic values obtained as heifers to the performance of cows with the highest genomic values is often employed in communication with breeders, such comparisons typically lack accompanying statistical analysis (Weigel et al. 2015; Bengtsson et al. 2020). Rather the economic significance of selection decisions based on ancestral information versus decisions made based on individual genomic evaluation is emphasized. Bengtsson et al. (2020) statistically tested the differences in correlations between breeding values and performance before comparing quartiles. In this work, the statistically significant correlations between breeding values and performance resulted in significant differences in the average adjusted phenotypes between the top and bottom herd quartiles. These differences were significantly higher for GBV than for parent averages. Protein yield stands out in this context; the increase in correlation after adding genomic information was smaller for this trait compared to other production traits. When all herds were analyzed together for this trait, Cohen’s *d* factor did not confirm that the increase in the difference between the top and bottom quartiles was significantly higher after adding genomic information. The reason for this exception is not clear. Although fat yield and protein yield have similar heritability and GBV reliability, protein yield exhibits lower variability, which may result in reduced phenotypic variation, which in turn can make it more challenging to demonstrate statistically significant differences.

By substituting PI with GBV, the relative change in differences between quartiles showed an increase of 64% for milk yield and 76% for fat yield, while in the study by Bengtsson et al. (2020), these figures were lower, equaling 42% and 47%, respectively. However, the differences in performance between the extreme quartiles were much smaller in this study. For milk yield, the differences were 363.9 kg for pedigree index and 600.9 kg for GBV, while in the Nordic study, they were 1061 kg and 1512 kg, respectively. For fat yield, the values in this study were 12.81 kg and 22.60 kg, whereas in the comparing one, they were higher and equaled 33 kg and 48 kg. Given that the milk production levels in both studied populations were similar: 9629 kg for Polish Holstein–Friesian and 9452 kg for Holstein in Nordic countries (Bentsson et al. 2020), it can be concluded that although analyzed GBV can help Polish farmers select animals with higher performance, it does so less effectively than in the Nordic population. Even larger differences in production traits between the highest and lowest quartiles were observed by Weigel et al. (2015). For milk yield, the difference was 2177 kg when cows were ranked by GBV, compared to only 1073 kg when ranked by their sire’s breeding value. It should be noted that these differences were observed in an analysis conducted within a single herd, where milking is performed three times a day and productivity exceeds 14,000 kg.

The differences between the top and bottom quartiles illustrate the potential benefits of selecting the best groups of animals. While these benefits were observed for SCC and stature, they were not apparent for the other traits. Furthermore, no significant differences were found between the extreme quartiles for udder and longevity. The explanations for these results are similar to those presented in the analysis of correlations between breeding values and performance. Additionally, it is worth noting that Bengtsson et al. (2020) found differences between the top and bottom quartiles for longevity of Holsteins, but did not find them for Red Dairy Cattle and Jersey breeds. It suggests that demonstrating the benefits of genotyping dairy cows across all traits and populations is not so easily accomplished.

Substantial GxE interaction within a breeding program is generally not anticipated, but it may occur, particularly when there are significant differences in management or climate conditions (Buckley et al. 2000; Fulkerson et al. 2008). Mulder et al. (2006) observed that even if the genetic correlation drops below 0.8 in certain scenarios, a single breeding program can still be more effective than operating two separate ones. Kolmodin et al. (2010) analyzed GxE interaction in Nordic Red dairy breeds and noted that genetic parameters change across different environments. In a recent study by Santana et al. (2023), which compared the performance of US Holstein cows in California and New England, the genetic correlation between production traits in the two environments did not fall below 0.92, and the ranking of the top 100 bulls showed a correlation no lower than 0.87. These results indicate that the presence of genotype-by-environment interaction is rather low and does not warrant consideration in national genetic evaluations. The average production level of a herd is often used as an indicator of the feeding level (Calus et al. 2002; Fikse et al. 2003; Hayes et al. 2016). In this study, differences between environments were indicated by average herd milk yield, showing that these differences impact how genetic merit translates into productivity. Fewer traits demonstrated that GBV outperformed PI in lower-producing herds. When analyzing herds with high and low productivity, estimates for the main fixed effects were similar, though the differences between classes were smaller in lower-producing herds (results not shown), which is a natural consequence of the general observation that lower productivity is typically associated with lower variability.

For production traits, stronger correlations between phenotype and breeding value were observed in higher-producing herds. Using GBV instead of PI had a relatively greater positive impact on correlations in lower-producing herds, suggesting that the benefit of using GBV to identify the best individuals is greater in less productive herds. However, the ratio between the average difference in PI or GBV and the average differences in adjusted phenotype was more favorable in higher-producing herds. This suggests that differences in breeding values translate more effectively into performance differences when animals are ranked by GBV, particularly in high-producing herds. In other words, breeders may realize greater benefits from having animals of higher genetic merit if they can provide an optimal environment for their cows. This does not imply that differences in breeding values are less relevant in low milk-producing herds, but rather that the expression of genetic potential may be more pronounced in better-managed environments. The observation that in a more productive environment, greater differences in performance can be seen between cows with different breeding values aligns with the findings of Fulkerson et al. (2008), who modified the environment by increasing dietary supplementation with additional concentrates and found that cows’ response (performance of production traits) to concentrate level increased as genetic merit increased. The average number of genotyped females in lower-producing herds was markedly smaller than in those with more effective management. This, combined with the lower number of animals in these herds, may have resulted in less effective accounting for non-genetic effects and, consequently, weakened the relationships between breeding values and performance in this group of herds, thereby hindering the benefits of increased reliability of breeding values.

## Conclusions

This study demonstrated that both pedigree indices and genomic breeding values effectively translate into performance in Polish Holstein–Friesian cows. For the most economically important traits such as milk yield, fat yield, protein yield, somatic cell score, and stature, and to some extent to protein yield genomic breeding values proved to be better indicators compared to pedigree indices. Given that these traits are weighted within the economic index to maximize profitability, it is advisable to utilize index values calculated based on genomic breeding values.

For other traits such as udder health, feet and legs, heifer conception rate, and longevity, it was not possible to show that adding genomic information makes a statistically significant difference. This difficulty can be attributed to the relatively low reliability of breeding values for these traits, discrepancies between the definition of breeding value and observed performance, phenotypic data quality, and the structure of the analyzed datasets. Hence, enhancing the reliability of breeding values for functional traits remains a key objective for future research and breeding programs.

While incorporating genomic information into breeding value estimations results in greater differences in milk yield performance in both low- and high-producing herds, it is noteworthy that in high-producing herds, this increase also extends to fat and protein yields. This finding underscores the importance of providing optimal environments for cows to fully realize the genetic potential indicated by GBV, thereby maximizing the benefits of genomic selection in dairy breeding programs.

## Data Availability

Data may be made available upon arrangement with its owner.
